# Hepatitis C Virus Proteins Activate NRF2/ARE Pathway by Distinct ROS-Dependent and Independent Mechanisms in HUH7 Cells

**DOI:** 10.1371/journal.pone.0024957

**Published:** 2011-09-13

**Authors:** Alexander V. Ivanov, Olga A. Smirnova, Olga N. Ivanova, Olga V. Masalova, Sergey N. Kochetkov, Maria G. Isaguliants

**Affiliations:** 1 Engelhardt Institute of Molecular Biology, Russian Academy of Sciences, Moscow, Russia; 2 Department of Molecular Biology, Tumor and Cell Biology, Karolinska Institutet, Stockholm, Sweden; 3 D.I. Ivanovsky Institute of Virology, Moscow, Russia; Duke University School of Medicine, United States of America

## Abstract

Hepatitis C virus (HCV) is a highly pathogenic human virus associated with liver fibrosis, steatosis, and cancer. In infected cells HCV induces oxidative stress. Here, we show that HCV proteins core, E1, E2, NS4B, and NS5A activate antioxidant defense Nrf2/ARE pathway *via* several independent mechanisms. This was demonstrated by the analysis of transient co-expression in Huh7 cells of HCV proteins and luciferase reporters. Expression, controlled by the promoters of stress-response genes or their minimal Nrf2-responsive elements, was studied using luminescence assay, RT-qPCR and/or Western-blot analysis. All five proteins induced Nrf2 activation by protein kinase C in response to accumulation of reactive oxygen species (ROS). In addition, expression of core, E1, E2, NS4B, and NS5A proteins resulted in the activation of Nrf2 in a ROS-independent manner. The effect of core and NS5A was mediated through casein kinase 2 and phosphoinositide-3 kinase, whereas those of NS4B, E1, and E2, were not mediated by either PKC, CK2, PI3K, p38, or ERK. Altogether, on the earliest stage of expression HCV proteins induced a strong up-regulation of the antioxidant defense system. These events may underlie the harmful effects of HCV-induced oxidative stress during acute stage of hepatitis C.

## Introduction

Hepatitis C virus (HCV) is a human pathogen which has infected 2–3% population worldwide [1]. In most cases HCV infection develops into chronic disease often manifested by liver steatosis and fibrosis, as well as non-liver diseases such as cryoglobulinemia, glomerulonephritis and others (for example, see [2], [3], [4] and references herein). HCV is an oncogenic virus strongly involved in the induction of hepatocellular carcinoma (HCC) [5] and possibly also non-Hodgkin lymphoma [6], driven by a complex yet incompletely understood pattern of virus-host interactions.

HCV replication induces oxidative stress, a phenomenon common in many chronic liver diseases [7], [8]. This stress contributes to insulin and interferon resistance, disorders of iron metabolism, liver fibrosis and HCC [9], [10], [11], [12], [13]. Specifically, virus nucleocapsid (core) and nonstructural NS5A proteins elevate the levels of reactive oxygen species (ROS) through alteration of calcium homeostasis [14], [15]. In addition, HCV proteins can induce NADPH oxidase 4 (Nox4) launching yet another mechanism of ROS formation [16], [17]. Finally, the glycoproteins E1 and E2 and the transmembrane protein NS4B induce ER stress and unfolded protein response [18], [19], which has been linked to ROS generation by activation of ER oxidoreductases [20].

Normally, ROS are neutralized by the low-molecular weight antioxidants, and “phase II detoxifying enzymes” [21]. Expression of phase II as well as of the enzymes of antioxidant biosynthesis (and also of “phase III efflux transporters”) is mainly regulated by NF-E2-related factor 2 (Nrf2). Nrf2 recognizes a conserved antioxidant response element (ARE) within the promoters of the responsive genes [22], [23]. Regulation of Nrf2 activity is mediated by its subcellular localization. In the absence of stress, the transcription factor is sequestered in the cytoplasm by its partner Keap1 [22], [24]. Phosphorylation of Nrf2 leads to their dissociation and subsequent translocation of Nrf2 to the nucleus [22], [23]. Nrf2 phosphorylation is mediated by protein kinase C (PKC), phosphoinositide-3-kinase (PI3K), mitogen-activated protein kinases (p38 and ERK1/2), and/or casein kinase 2 (CK2) [24], [25], [26], [27], [28]. The input of each of the kinases in Nrf2 activation depends on the cell type and stress stimuli.

The aim of this work was to study oxidative stress induction by individual HCV proteins at the earliest stage of their expression, and the involvement of Nrf2/ARE pathway in the reaction to this stress. This field was completely blank until two very recent but contradictory publications on the regulation of Nrf2/ARE system in HCV infected cells [26], [29]. Burdette et al revealed that HCV replication in HCV cell culture system (HCVcc) is accompanied by activation of Nrf2/ARE pathway which protects cells from oxidative stress-induced apoptosis [26]. In the same system, an independent study of Carvajal-Yepes et al evidenced a suppression of Nrf2 activation [29]. This study suggested that the down-regulation was due to core and NS3, their combined action causing the delocalization of small Maf proteins from the nucleus not allowing the formation of active Nrf2/Maf heterodimers [29]. Both papers described the consequences of HCV replication in cultured cells, the cooperative effect generated by all viral proteins 2 to 6 days postinfection, with no obvious reasons for the discrepancy between the results. The field clearly requires further mechanistic studies.

Here, we present a detailed overview of the oxidative stress induction with activation of Nrf2/ARE system by individual HCV proteins during the earliest stage of their expression. We have observed that five HCV proteins, namely, core, E1, E2, NS4B, and NS5A, can both induce elevated ROS levels and activate Nrf2/ARE pathway. The activation, which is partially ROS-independent, involves different protein kinases. On the overall, the study unveils the input of individual HCV proteins in induction and regulation of the oxidative stress response.

## Results

### Plasmid construction and characterization

To study the effects of HCV proteins on cellular defense system against oxidative stress, we constructed a set of plasmids expressing core, E1, E2, p7, NS2, NS3, NS4A, NS4B, NS5A, and NS5B proteins of HCV genotype 1b derived from Con1 or highly homologous 274933RU strains. The resulting plasmids were transfected into Huh7 cells. Transient protein expression was confirmed by SDS-PAGE and Western blotting (Fig. S1).

The quantification of expression by imaging of immunoblots using protein-specific antibodies demonstrated that accumulation of proteins started 16–20 h and reached a plateau at 30–36 h posttransfection (Fig. S2A and data not shown). The strongest differences were observed between the expression of NS5A, and NS5B, and HCV core (Fig. S2A). NS5A and NS5B were expressed at similar levels (600–700 fg per transfected cell at 30 h posttransfection), whereas the amount of core protein was significantly lower (appr. 100 fg per cell).

To study the influence of HCV proteins on regulation of expression of phase II detoxifying enzymes, we constructed a pP-ARE reporter plasmid containing a luciferase gene under the control of SV40 promoter modified to include the minimal ARE of one of the phase II detoxifying enzyme genes, human NAD(P)H:quinone oxidoreductase (Nqo1). The resulting plasmid directed the expression of luciferase in response to oxidative stress induced by treatment of cells with *tret*butylhydroquinone (tBHQ) or hydrogen peroxide (H_2_O_2_), which confirmed the functionality of ARE element (Fig. S3A).

### Five HCV proteins induce oxidative stress in Huh7 cells

**Figure 1:**
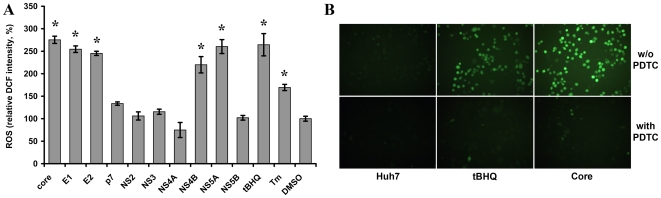
Effect of transient expression of HCV proteins on accumulation of reactive oxygen species (ROS). Transient expression of HCV core, E1, E2, NS4B, and NS5A proteins in Huh7 cells induced oxidative stress registered by ROS formation detected by fluorometry of DCF-DA-stained cells (**A**). Alternatively, ROS were visualized by fluorescence microscopy, as is shown for cells expressing core protein or treated with tBHQ or/and 40 µM ROS scavenger PDTC (**B**). Cells treated with 100 µM tBHQ (**A,B**) or Tm (**A**) are given as positive and DMSO-treated as negative controls. Error bars indicate SD. *P<0.01 versus DMSO (Tukey-Kramer test).

The role of individual HCV proteins in induction of oxidative stress was studied by measuring levels of ROS triggered by treatment of cells with oxidation-sensitive dye dichlorofluoresceine diacetate (DCF-DA). Induction of ROS led to an increase of fluorescence registered by microplate fluorimetry or fluorescence microscopy (Fig. 1). P7, NS2, NS3, NS4A, and NS5B had no effect on ROS levels (Fig. 1A). Expression of core and NS5A proteins induced considerable ROS production, in lines with their earlier described capacity to cause oxidative stress [14], [15]. The effect of these proteins was observed starting from 18–20 h after transfection (Fig. S2B) and was specific to these two proteins, since NS5B expressed at similar levels did not affect cell redox status. Similar effects were induced by NS5A and core (Fig. 1A) despite the latter being expressed at 6–7 fold lower level (Fig. S2A) indicating that core was a stronger ROS inducer. Surprisingly, expression of E1, E2, and NS4B proteins, not earlier involved in oxidative stress induction, also elevated ROS levels. In all these cases, induction of ROS was completely inhibited by pretreatment of cells with ROS scavenger, pyrollidine dithiocarbamate (PDTC) (Fig. 1B and data not shown).

### Effect of HCV proteins on ARE-dependent expression of luciferase

**Figure 2:**
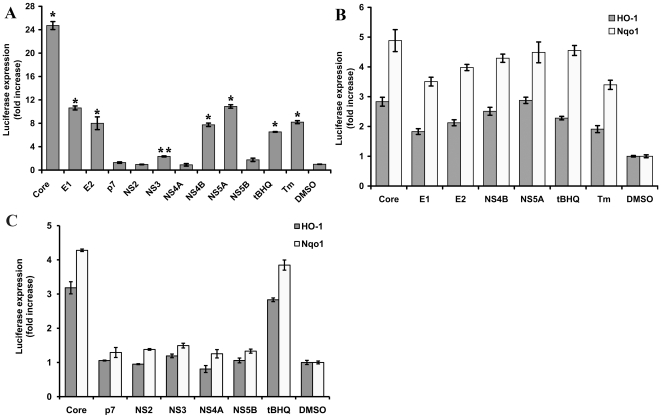
Impact of HCV proteins on ARE-dependent luciferase expression. Histograms of the relative luciferase activities detected for Huh7 cells transfected with plasmids encoding HCV protein and reporter plasmids bearing minimal ARE (*P<0.01 versus DMSO; **P = 0.03 versus DMSO; Tukey-Kramer test) (**A**) or the full-length promoters of HO-1 or Nqo1 genes (**B** and **C**) in the presence of HCV proteins expressed transiently: HCV proteins activating (**B**) or not influencing (**C**) transcription from HO-1 and Nqo1; HCV core is given as positive control of transcription activation. Error bars indicate SD.

The influence of HCV proteins on transcription of ARE-dependent genes was first studied using ARE-regulated luciferase reporter plasmid (pP-ARE). Its co-transfection with each of the plasmids expressing individual HCV proteins revealed that core, E1, E2, NS4B, and NS5A strongly stimulated luciferase expression (Fig. 2A). A weak effect on ARE-dependent transcription was also induced by the expression of NS3 (Fig. 2A). Surveys of luciferase activity done during two days posttransfection revealed that ARE-regulated expression of luciferase started 18 h and reached plateau 28–30 h post-transfection (Fig S2C). As in case of ROS induction, the difference in ARE-luciferase activation levels was not due to difference at the levels of HCV protein expression but was protein-specific since NS5B expressed to the same level as NS5A (and 6–7-fold more efficiently than core) did not affect the luciferase expression (Fig. 2A and S2C). Thus, we have shown that HCV proteins that induce oxidative stress, also up-regulate the luciferase expression from an ARE-containing promoter.

Maximum luciferase induction was achieved by HCV core. However, this protein also up-regulated reporter expression from a basal SV40 promoter of pGL3-promoter vector (Fig. S4A) as was described earlier [30]. In the case of pP-ARE, the effect of core protein was partially mediated through ROS, since treatment of cells with PDTC inhibited induction of SV40-ARE controlled reporter, while in case of SV40-controlled reporter it was ROS-independent (Fig. S4B). Other HCV proteins had no effect on the SV40-directed luciferase expression.

To confirm specific effects of HCV proteins on ARE-dependent transcription, the experiment was repeated using the native ARE-containing promoters of Nqo1 and heme oxygenase 1 (HO-1) genes (plasmids Nqo1-luc and pHOGL3/9.1, respectively). All HCV proteins that enhanced ARE-regulated transcription from pP-ARE, also stimulated luciferase expression from Nqo1 and HO-1 promoters (Fig. 2B). In contrast, p7, NS2, NS3, NS4A, and NS5B did not affect the activity of Nqo1 and HO-1 promoters (Fig. 2C). This experiment demonstrated the specific activity of HCV proteins, particularly on the transcription of ARE-responsive genes. The lower activation in case of HO-1 as compared to Nqo1 promoter, could result from the influence of other regulatory elements or from a non-optimal ratio between plasmid expressing HCV proteins and reporter vectors in the transfection.

### Effect of HCV proteins on induction of phase II detoxifying enzymes

**Figure 3:**
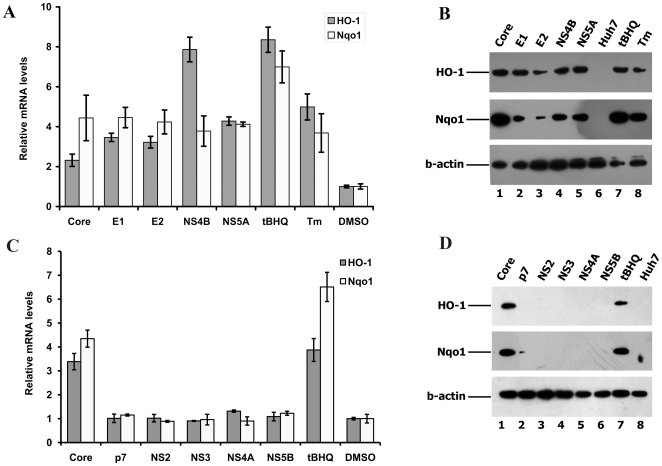
Up-regulation of HO-1 and Nqo1 gene expression by HCV proteins. Histograms of the relative ho-1 and nqo1 mRNA levels in Huh7 cells expressing HCV proteins, as quantified by RT-qPCR (Error bars indicate SD) (**A** and **C**). Western-blot analysis of the expression of HO-1 and Nqo1 of samples presented in panels A (panel **B**) and **C** (panel **D**). In both panels, Huh7 cells treated with oxidative stress inducer (tBHQ) or ER stress inducer (Tm) are given as positive and DMSO-treated cells as negative controls, and b-actin was used as an internal control. On panels **C** and **D** HCV core was also used as a positive control.

The next step of the study was to confirm the effects of HCV proteins which induced ARE-regulated transcription, on the expression of phase II detoxifying enzymes HO-1 and Nqo1. For this, we transfected Huh7 cells with plasmids expressing HCV proteins, or treated untransfected cells with tBHQ or Tm. Nqo1 and HO-1 mRNA levels were estimated using quantitative RT-PCR and protein levels, using Western-blot. HCV core, E1, E2, NS4B, and NS5A proteins as well as the control stress inducers up-regulated the transcription of HO-1 and Nqo1 genes (Fig. 3A). Accordingly, HO-1 and Nqo1 protein levels were significantly higher compared to these in the non-transfected Huh7 cells or cells transfected with empty pcDNA3.1(+) vector (Fig. 3B). HCV core initially inhibited transcription of HO-1 gene (see Fig. S5 of effect 31 h post transfection) but activated HO-1 expression at a later time point (Fig. 3A, 40 h post transfection). Expression of other HCV proteins (p7, NS2, NS3, NS4A, and NS5B) did not alter HO-1 and Nqo1 mRNA or protein levels (Fig. 3C,D). The expression data was concordant with the data on the effects if HCV proteins on ARE-regulated reporter expression (Fig. 2). Therefore, we conclude that HCV proteins, which induce oxidative stress, can also activate the expression of phase II detoxifying enzymes.

### HCV proteins activated ARE-regulated gene expression through ROS-dependent and –independent phosphorylation of Nrf2 that lead to its nuclear translocation

**Figure 4:**
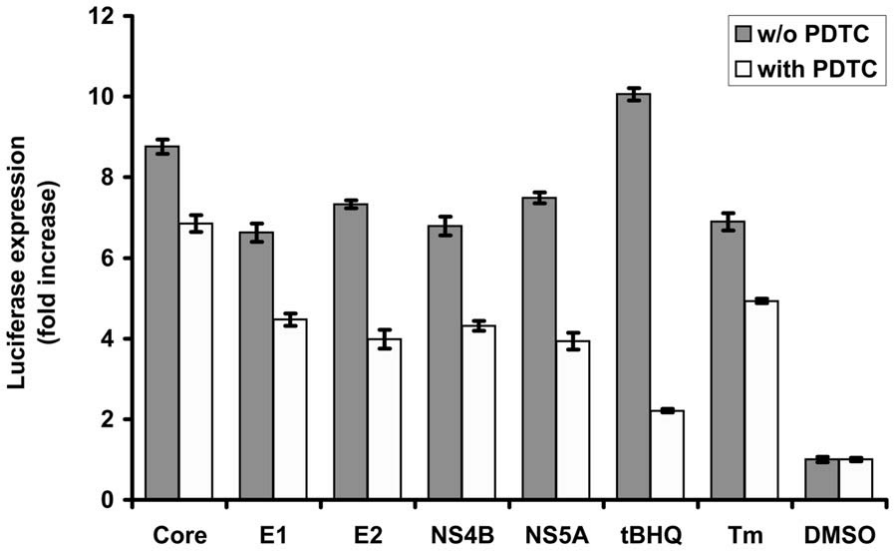
Effect of ROS scavenger PDTC on ARE-dependent luciferase expression induced by HCV proteins. Luciferase activity was measured in lysates of Huh7 cells expressing core, E1, E2, NS4B, or NS5A proteins, pre-treated with PDTC. The cells treated with oxidative stress inducer (tBHQ) or ER stress inducer (Tm) are given as positive and DMSO-treated cells as negative controls. Error bars indicate SD.

Next, we have investigated the mechanisms by which HCV proteins modulate ARE-regulated transcription, specifically if ARE-regulated luciferase expression is ROS-dependent. Treatment of cells with PDTC diminished tBHQ-induced luciferase expression by almost five-fold indicating a high degree of ROS-dependence (Fig. 4). In contrast, in cells expressing HCV proteins, ARE-regulated reporter expression was reduced by ROS scavenger only partially (by 1.4–1.9 fold), and in case of core, only 1.3 fold. This indicated that ARE-regulated transcription in cells expressing HCV proteins occurred also in a ROS-independent manner. Interestingly, a similar PDTC effect was observed for Huh7 cells treated with a control ER stress inducer tunicamycin (Tm) (Fig. 4).

**Figure 5:**
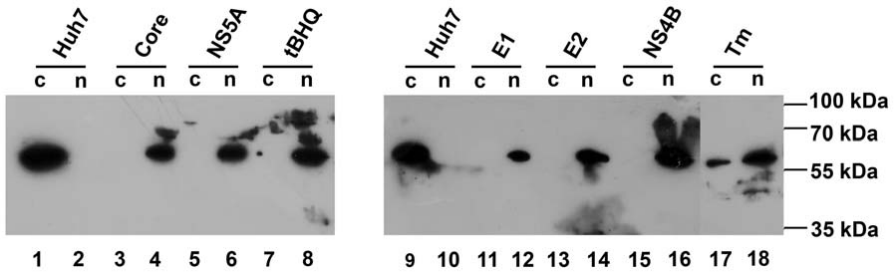
Influence of HCV proteins on Nrf2 subcellular localization. Nrf2 localization was determined by separation of cytoplasmic (c) and nuclear (n) protein fractions as described in “Materials and Methods” section with subsequent detection of the transcription factor by Western blot analysis. tBHQ and Tm were used as control stress inducers.

Next, we have investigated the involvement in expression of ARE-dependent genes of Nrf2 transcription factor. Activation of Nrf2 is achieved by its translocation from the cytoplasm to the nucleus [21]. So we separated cytoplasmic and nuclear protein fractions by the standard procedures (see “Materials and Methods”) and estimated the amount of Nrf2 in each by Western blotting. Expression of core, E1, E2, NS4B, and NS5A proteins caused Nrf2 translocation from cytoplasm into the nucleus (Fig. 5). Similar effects were observed for the control stress inducers tBHQ and Tm. This verified that activation of the ARE-dependent genes was concomitant with the activation of Nrf2.

**Figure 6:**
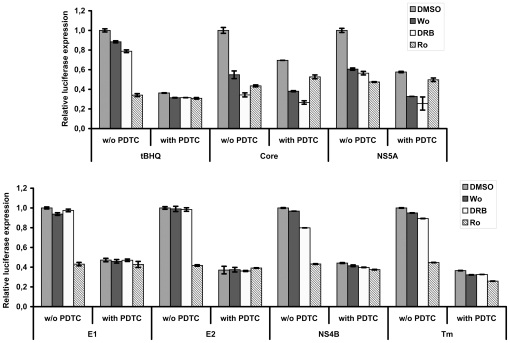
Influence of protein kinases inhibitors on ARE-dependent luciferase expression induced by HCV proteins. Histograms of relative luciferase activity in Huh7 cells expressing HCV core, E1, E2, NS4B, or NS5A proteins, treated with commercially available inhibitors of PI3K (wortmannin, Wo), CK2 (DRB), or PKC (Ro 31-8220, Ro). In a parallel experiment the cells were pre-treated with ROS scavenger PDTC. The control cells were treated with DMSO. Error bars indicate SD.

Nrf2 translocation is regulated by the following enzymes: PKC, PI3K, CK2, p38 and ERK1/2 mitogen-activated protein kinases, and PERK [24], [25], [26], [27], [28], [31]. Our next step was to reveal which cellular protein kinases are involved in ARE-luciferase gene induction by individual HCV proteins. For this, we have used the following inhibitors: wortmannin for PI3K, Ro31-8220 for various isoforms of PKC, Sb239063 for p38 MAPK, Pd98.059 for ERK, and DRB for CK2. The inhibitors were added 18 h posttransfection, and cells were incubated in their presence for additional 10 h. The results are summarized on Fig. 6 (for complete data see Fig. S6). The inhibitors had little effect on the basal luciferase expression (Fig. S6A). Under tBHQ-induced oxidative stress, ARE-luciferase induction required the activity of PKC, although an inhibitor of CK2 also caused a small but statistically significant effect (Fig. 6 and Fig. S6B). Noteworthy, PKC was activated by ROS, since the corresponding inhibitor was inactive in cells treated with PDTC. In cells expressing core and NS5A, stimulation of ARE-dependent luciferase transcription was mediated *via* three kinases: PKC, PI3K, and CK2 (Fig. 6 and Fig. S6C,D). Wortmannin and DRB inhibited luciferase expression both in the absence and in the presence of antioxidant PDTC, whereas Ro31-8220 exhibited the activity only in the absence of PDTC. This indicated that PKC-mediated activation was ROS-dependent (as in the case of tBHQ), whereas two other kinases were activated in a ROS-independent manner. In cells expressing E1, E2, or NS4B or treated with Tm, luciferase expression was up-regulated by the ROS-PKC pathway; other inhibitors tested did not exhibit any notable ROS-dependent or independent effects (Fig. 6 and Fig. S6E-H). This indicated that the kinases governing ROS-independent Nrf2 activation by these proteins remain to be found.

**Figure 7:**
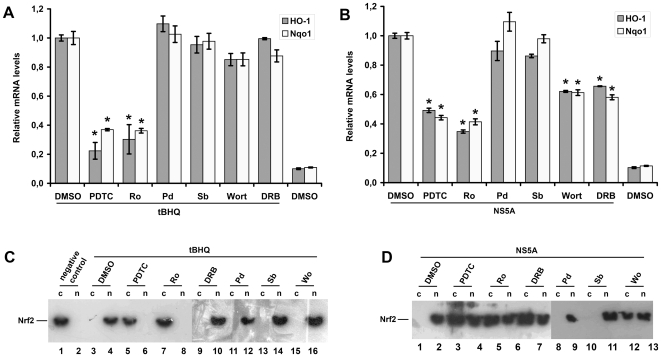
Effect of protein kinase inhibitors and PDTC on HO-1/Nqo1 gene expression, and Nrf2 localization. (A,B). Histograms of relative HO-1 and Nqo1 mRNA levels in Huh7 cells treated with tBHQ (**A**) or expressing NS5A protein (**B**), treated with the inhibitors of PI3K (wortmannin, Wo), p38 (SB 239063, Sb), ERK1/2 (PD98,059, Pd), CK2 (DRB), or PKC (Ro 31-8220, Ro), or with ROS scavenger PDTC. Quantification of HO-1 and Nqo1 mRNA levels was performed by RT-qPCR using b-actin as a loading control (*P<0.01 versus DMSO, and tBHQ-treated cells or DMSO-treated cells expressing NS5A, respectively). (**C,D**). Western blot analysis of Nrf2 subcellular localization in the same samples used in panels (**A**) and (**B**).

Next, we have studied the effects of protein kinase inhibitors on the expression of phase II detoxifying enzymes HO-1 and Nqo1 and on the localization of Nrf2 on the example of NS5A protein. We added the same inhibitors to the NS5A-expressing cells and quantified the levels of HO-1 and Nqo1 mRNA. Here, as above, the effect of the control inducer of oxidative stress tBHQ on the transcription of HO-1 and Nqo1 genes was mediated solely by PKC (Fig. 7A), while the effect of NS5A protein expression was sensitive to the inhibitors of PKC, CK2, and PI3K (Fig. 7B). In lines with this, Nrf2 nuclear translocation in NS5A-expressing cells was partially blocked by PKC, PI3K, and CK2 inhibitors (Fig. 7D), while in the tBHQ-treated cells it could be completely prevented only by the PKC inhibitor (Fig. 7C). In a separate experiment, cells were treated with PDTC to reveal if the activation occurred also *via* ROS-independent mechanism. Indeed, PDTC completely prevented Nrf2 translocation in case of tBHQ, whereas in case of NS5A the inhibition was only partial (Fig. 7C,D). Thus, NS5A was involved in the activation of cellular antioxidant defense system through both ROS-dependent and independent pathways.

## Discussion

Since its discovery, HCV-induced oxidative stress has been implicated in the development of a variety of virus-associated liver injuries and metabolic disorders [9], [10], [11], [12], [13]. Very little, however, was known about the exact molecular mechanism(s) behind the induction of HCV-induced oxidative stress and the status of respective defense system, strongly motivating such studies. The aim of this work was to investigate the molecular mechanisms underlying oxidative stress and stress response induced by the individual HCV proteins. Our work on the effects of the individual proteins developed in parallel to the studies of the effects of the whole HCV proteome. Recently, Burdette et al reported activation of this pathway in response to an oxidative stress induced by HCV replication in the infectious cell system and, as a consequence, the induction of high levels of phase II enzymes [26]. Very recently, in the same system, Carvajal-Yepes et al reported a contradictory data on the suppression of Nrf2/ARE pathway [29], indicating that a lot in this field is yet unknown.

The cellular machinery which protects cells against the harmful effects of reactive oxygen species has been investigated by many groups using various models that often generated contradictory results. Most researchers agreed that the expression of several antioxidant genes in HCV-infected cells is up-regulated as compared to the naïve Huh7 cells [32]. However, the actual levels of enzymes of glutathione homeostasis or of metallothioneins were reported to be either higher or lower than in uninfected/untransfected cells [32], [33], [34]. These and similar discrepancies could be due to the use of different experimental systems (stable cell lines vs. HCV infectious culture), or analysis technique (qPCR, Western-blotting and proteomic analysis vs. Affymetrix Gene chips). However, the data on Nrf2/ARE regulation in HCVcc system [26], [29] was generated in one and the same system (JFH-1 strain) using similar methodological approaches which does not explain the controversy in the results obtained. Our study, elucidating the role of individual viral proteins, fills this gap.

We found that five HCV proteins, namely core, E1, E2, NS4B, and NS5A, stimulate generation of ROS and activate Nrf2-mediated gene transcription. The experiments on the activation of Nrf2/ARE pathway activation using various methods (reporter assays, RT-qPCR, Western blotting) gave concordant results. Activation observed in response to core, E1, E2, NS4B, and NS5A was specific. Other HCV proteins, such as p7, NS2, NS3, NS4A or NS5B, did not alter the activity of HO-1 or Nqo1 promoters or of the minimal ARE element, and had no effect on HO-1 and Nqo1 mRNA and protein levels. The effects of core, E1, E2, NS4B, and NS5A were observed as early as 18–20 h and lasted for ≥40 h post-transfection. An exception was the expression of HO-1: transcription and translation of this gene was first suppressed, and then activated (31 and 40 h post-transfection, respectively). Surprisingly, activation of Nrf2/ARE pathway in case of all these proteins did not result exclusively from the oxidative stress signaling, but also from a ROS-independent process. We have shown that ROS-dependent mechanism of activation of ARE-dependent genes involves phosphorylation of Nrf2 transcription factor by PKC, whereas a ROS-independent effect is mediated by CK2 and PI3K. Noteworthy, HCV core acted as a potent inducer of oxidative stress and activator of the defense system even when present in small amounts (due to low levels of transient expression). Altogether, this demonstrates that HCV proteins trigger activation of Nrf2/ARE pathway *via* several (parallel) mechanisms from the very on-set of their expression.

The activation of Nrf2/ARE pathway appears to be a common property of HCV of different genotypes, as was demonstrated for HCV 1b-derived proteins (here) and for the HCV 2a proteome (by Burdette et al). Our data specify that this activation and the concomitant oxidative stress may arise from the expression of five viral proteins, namely core, E1, E2, NS4B, and NS5A. We found that stress response starts early, and Burdette et al followed it up to day 6 post infection. Altogether, these results do not support the down-regulation of ARE-dependent genes by HCV proteins [33]. Neither can we refute the data of Carvajal-Yepes et al [29], since the suppression they revealed was due to the combined effect of HCV proteome, namely of core and NS3, whereas we have worked with individual HCV proteins. These controversy motivated us to perform additional experiments in which we have studied the effects on Nrf2/ARE pathway of co-expression of HCV nonstructural proteins NS3 to NS5B. We revealed that their simultaneous expression also activated Nrf2 (data not shown), indicating that Maf relocalization-driven inhibition may dependon the presence of other/structural HCV proteins, such as core. We are currently verifying this possibility with a set of experimental approaches employed here but using HCV protein combinations.

Both our and Burdette et al [26] data indicate that during the acute phase of HCV infection the levels of antioxidants and of the phase II antioxidant enzymes may be elevated. The hazardous processes triggered by oxidative stress occur in the cell in the first few hours after the on-set of stress reactions (for example [35], [36]). At the early stages, the infected cells may gain the capacity to protect themselves against the stress insult by enhancing the expression of antioxidant genes. The latter might be unfavorable for propagation of HCV that elaborates strategies of reducing oxidative stress and/or oxidative stress-response. If this is true, the study of Carvajal-Yepes et al may present an example of such negative feedback, i.e. the reduction of expression of Nrf2-dependent genes after the initial activation. This falls in lines with the increased levels of oxidized glutathione and of other oxidative stress markers in blood; normal or suppressed levels of antioxidants and phase II enzymes [37], [38]; and elevated sMaf levels in the liver tissues of chronic hepatitis C patients [29]. It also correlates with the expression of another Nrf2-dependent gene *mrp2* encoding a hepatocyte transporter which plays an important role in biliary excretion of bilirubin and glutathione [39], [40]. The expression of *mrp2* is up-regulated in cells harboring an HCV replicon [39], but is suppressed in the liver of chronic HCV carriers [41]. Unfortunately, we were unable to find any published data on the levels of antioxidants in the acute HCV infection that would support this concept. Further studies of the changes in Nrf2/ARE pathway status during transition from acute to chronic infection are urgently required.

PKC isoforms are responsible for Nrf2 activation in several cell types including hepatoblastoma HepG2 [27], [42]. Our data demonstrate that they are involved also in the hepatocytes expressing individual HCV proteins indicating that their effect on PKC is not specific to the protein. On contrary, both NS5A [43] and apparently also core [44] can directly activate PI3K, their effects on Nrf2 translocation are, therefore, protein-specific. The mechanism of casein kinase 2 activation in cells expressing core or NS5A proteins is unclear. However, it has been reported that the activity of this kinase can be affected by several factors including alteration of calcium homeostasis [45], a known effect induced by both core and NS5A proteins [46]. In this study we did not reveal which kinases are involved in the ROS-independent Nrf2 activation in response to E1, E2, and NS4B-induced stress except for this not being PKC, CK2, PI3K, p38, or ERK (Fig. 6 and Fig. S6E-G). A possible option is the PKR-like endoplasmic reticulum kinase (PERK) [31], but experimental proof of this option is hampered by the lack of commercially available PERK inhibitors.

It is worth noting that our results differ from those obtained by Burdette et al [26] who found that activation of Nrf2/ARE pathway by HCV proteins is mediated through p38 and JNK MAP kinases. These differences could be due to several factors. One of them is the use of different systems (expression of individual proteins of genotype 1b *vs.* infectious HCV of genotype 2a). Also it could be due to a different time point for the analysis: early in our case versus 4 days post-infection in case of [26]. Thirdly, Burdette et al used PKC inhibitor Go6976 capable of blocking only the classical enzyme isoforms, not implicated previously in Nrf2 activation. In contrast, we have used a pan-PKC inhibitor Ro31-8220 and thus were able to detect the effects of the whole PKC family.

The biological consequences of Nrf2/ARE pathway regulation are multifacetted. Oxidative stress reactions, if not alleviated, quickly (within a few hours after stress induction) lead to a cell death by apoptosis (see, for example, [35], [36]). Indeed, when we inhibited Nrf2/ARE activation with PKC inhibitor, or a combination of PI3K and CK2 inhibitors 48 h post-transfection, the cells underwent apoptosis, while cell growth was not altered in the inhibitor-treated cells transfected with empty vector used as a control (data not shown). Clearly, the activation of antioxidant defense system during HCV protein expression, as in acute HCV infection, can mitigate harmful effects of oxidative stress protecting affected cells. However, activation of Nrf2/ARE pathway may also alleviate the ROS-mediated inhibition of virus replication [47] (actually favoring viral replication) and trigger cell transformation [26]. This suggests that the use of antioxidants to protect infected cells during acute hepatitis C may not be advisable. At the same time, possible inactivation of Nrf2/ARE pathway during the chronic stage would justify treatment of patients with antioxidants to protect from the long-term stress effects. Indeed, a pilot trial revealed that in patients with elevated liver enzyme levels treatment with a combination of antioxidants helps to alleviate necro-inflammatory events and normalize liver enzymes [48].

In summary, we have specified the molecular mechanisms of regulation of Nrf2/ARE pathway by individual HCV proteins during the initial steps of their expression in Huh7 cells. These molecular events may contribute to the overall effect of virus replication on the antioxidant defense system during the acute stage of the disease. Further studies are required to gain a systematic understanding of the in-put of oxidative stress into the pathogenesis of HCV infection and HCV-related disorders.

## Materials and Methods

### Reagents

Lipofectamine 2000 was purchased from Invitrogen (Carlsbad, CA, USA), Dulbecco's modified Eagle medium (DMEM), nonessential amino acid solution, and antibiotics for cell cultures were from PanEco (Moscow, Russia). Fetal calf serum (FCS) was obtained from HyClone (Logan, UT, USA). 2′,7′-Dichlorofluorescein diacetate (DCFH-DA) and ammonium pyrrolidine dithiocarbamate (PDTC), protein kinase inhibitors 5,6-dichloro-1-beta-Dribofuranosylbenzimidazole (DRB), Wortmannin, SB 239063, Ro 31-8220, and PD98,059, protease inhibitor cocktail as well as other chemicals were purchased from Sigma (St. Louis, MO, USA), unless otherwise noted. Antibodies to Nrf2 (ab31163), Nqo1 (ab28947), HO-1 (ab13248), HCV protein E2 (ab20852-100), HCV protein E1 (ab21306-100), HRP-conjugated anti-rabbit and anti-mouse secondary antibodies were obtained from Abcam (Cambridge, UK), monoclonal antibodies to core (clone d4), NS4A (clone 3F12) and NS4B (clone 6B11) proteins were previously described [49], [50]. Antibodies to β-actin (A1978 clone AC-15) were obtained from Sigma (St. Louis, MO, USA), Hybond-ECL membrane was supplied by GE Healthcare (Chalfont St. Giles, UK), ECL detection and Nuclear and Cytoplasmic Extraction Reagents (NE-PER kit) reagents were obtained from Thermo Scientific (Rockford, IL, USA). qPCRmix-HS master mix was from Evrogen (Moscow, Russia). Taq and Pfu DNA polymerases, restriction enzymes and T4 DNA ligase were from Fermentas (Vilnius, Lithuania) or from Sibenzyme (Novosibirsk, Russia). All unmodified oligonucleotides were synthesized by Lytech (Moscow, Russia), and Taqman probes for qPCR were obtained from Syntol (Moscow, Russia). Huh7 cells were a kind gift of Prof. R. Bartenschlager (Heidelberg University, Germany) [51].

### Plasmid constructs

Plasmids pCMVcore and pCMVNS3 expressing core protein and NS3-protease of HCV isolate 274933RU (GenBank: AF176573) were described in [52]. Genes of E1, E2, p7 NS2, and NS4A nonstructural proteins were amplified with pairs of forward and reverse primers (Table S1) from plasmids pEsNS-2-217 or p4216-5647 (HCV isolate 274933RU), whereas genes of NS4B, NS5A, and NS5B proteins were amplified from plasmid pI341*/*NS3*-*3′*/*LucUbiNeo-ET (HCV isolate Con1, GenBank: AJ238799). The obtained DNA fragments were digested with restriction nucleases (Supplementary table S1) and cloned into pcDNA3.1(+) vector (Invitrogen) to give target expression plasmids (pcDNA-E1 to pcDNA-NS5B). To construct the pP-ARE reporter plasmid bearing ARE of human Nqo1 gene, oligonucleotides hARE-For and hARE-Rev were phosphorylated with T4 polynucleotide kinase, annealed, and ligated into Kpn1 and NheI sites of pGL3-promoter vector (Promega, Madison, WI, USA).

Plasmids pLucNQO1 [53] and pHOGL3/9.4 [54] were a kind gift of Dr. R. Faraonio (Università di Napoli Federico II) and Dr. Traylor and Dr. Agarwal (The University of Alabama at Birmingham), respectively.

### Expression of HCV proteins in Huh7 cells

Human hepatoma Huh7 cells were maintained in 5% CO_2_ at 37°C in DMEM with 10% fetal calf serum, 2 mM glutamine, 50 U/ml penicillin and 50 µg/ml streptomycin. Twenty four hours prior to transfection, cells were seeded in 6-well plates at a density of 3×10^5^ cells/well into antibiotic-free DMEM with FCS and glutamine, and grown to attain 90–95% confluence. On the next day, for each well, 1 µg of pcDNA-derived plasmid was added to 250 µl of DMEM, 2 µl of Lipofectamine 2000 was added to another tube with 250 µl of DMEM, and both solutions were incubated at room temperature for 5 min, combined and kept for additional 45 min. The cells were incubated with the transfection complexes for 4 hours in serum-free medium which was replaced with DMEM supplemented with FCS and glutamine. Thirty-six hours after the cells were washed with phosphate-buffered saline (PBS) and cellular lysates were prepared by incubating in radioimmune precipitation (RIPA) buffer (50 mM Tris-HCl, pH 7.5, 150 mM NaCl, 1% NP-40, 0.25% sodium deoxycholate, 1 mM PMSF, 1x Protease Inhibitor Cocktail) for 30 min on ice. The lysates were denatured at 100°C for 5 min in sample buffer, then subjected to 12% SDS-PAGE and transferred onto a nitrocellulose Hybond-ECL membrane in 25 mM Tris, 192 mM glycine and 20% methanol, which was blocked with 5% nonfat milk in PBS for 1 h at room temperature (RT). Membranes were probed with the primary antibody: anti-Core antibody (2 µg/ml), anti-HCV protein E1 antibody (2 µg/ml), anti-HCV protein E2 antibody (2 µg/ml), anti-NS3 serum (1∶500), anti-NS4A antibody (1.6 µg/ml), anti-NS4B antibody (2 µg/ml), anti-NS5A serum (1∶300), anti-NS5B serum (1∶500), at 4°C overnight and washed twice for 10 min with PBST (PBS with 0.5% Tween-20) followed by incubation with secondary antibody: anti-mouse antibody (0.5 µg/ml) or anti-rabbit antibody (0.45 µg/ml) for 1 h at RT. After an additional washing step with PBST, immunoblots were visualized using ECL detection system.

### Reporter assays

The transfection was performed similarly to the described above with two modifications. Firstly, before transfection the cells were seeded in 24-well plates at a density of 8×104 cells grown to 80–95% confluence. Secondly, for each well, the transfection complexes were formed by a mixture of 0.8 µl Lipofectamine 2000 with 0.2 µg of pcDNA-derived plasmid, 0.4 µg of pSV-β-galactosidase control vector, and 0.2 µg of pGL3-promoter, pP-ARE, pLucNQO1 or pHOGL3/9.4 plasmids. Luciferase activity was measured twenty-eight hours after transfection using a luminometer (Turner Designs). Transfection efficiency was normalized on the basis of β-galactosidase activity. In order to determine which protein kinase is responsible for Nrf2 activation, 18 hours after transfection the cells were treated with inhibitors of PKC (2 µM Ro 31–8220), PI3K (1 µM Wortmannin), p38 (2 µM SB 239063), ERK (10 µM PD98,059), dissolved in DMSO, or CK2 (40 µM DRB), dissolved in ethanol.

In all experiments 100 µM tBHQ and 2 µM Tm if not stated otherwise were used as control inducers of the oxidative or ER stress, respectively. H_2_O_2_ (400 µM) was used as an alternative oxidative stress inducer only in the initial experiments on pP-ARE plasmid characterization since it was found to affect cell viability (Fig. S3B). In addition, when ROS influence on Nrf2 activation was investigated, antioxidant PDTC was added eighteen hours after transfection to 40 µM final concentration. Two hours later, the inhibitors were added to the antioxidant-containing medium.

### Measurement of reactive oxygen species

Intracellular reactive oxygen species (ROS) production was measured by epifluorescence. The Huh7 cells were transfected with plasmids, expressing HCV proteins as described above. The growth medium was removed 16, 18, 20, 22, 24 hours after transfection, the cells were incubated in medium containing 25 µM dichlorofluorescein diacetate (DCFH-DA) at room temperature for 30 min. The cells were washed 10 times with 500 µl of PBS. The fluorescence intensities (FLI) were measured in PBS (200 µl) using Plate CHAMELEON V reader (Hidex Ltd.) with excitation at 485 nm and with emission at 535 nm.

### Quantitative real-time reverse transcription (RT) – PCR

RNA was isolated from 5×10^5^ cells with PerfectPure RNA Cultured Cell kit (5Prime) and reverse transcribed using Reverse Transcription System (Promega) with random hexamer primer according to manufacturers' protocol. QPCR was performed using IQ5 Real-Time PCR Detection System (BioRad). Primer and probe sequences were as follows: HO-1: 5′-CCAGCAACAAAGTGCAAGATTC-3′ (sense primer), 5′-TCACATGGCATAAAGCCCTACAG-3′ (antisense primer), 5′-[Cy5]-TCTCCGATGGGTCCTTACACTCAGCTTTCT-[BHQ2]-3′ (probe); Nqo1: 5′-GTCATTCTCTGGCCAATTCAGAGT-3′ (sense primer), 5′-TTCCAGGATTTGAATTCGGG-3′ (antisense primer), 5′-[CY5]-ACTGACATATAGCATGGGCACACTCCAGC-[BHQ2]-3′ (probe); β-actin: 5′-GATCATTGCTCCTCCTGAGC-3′ (sense primer), 5′-ACTCCTGCTTGCTGATCCAC′ (antisense primer), 5′- [R6G]-CTCGCTGTCCACCTTCCAGCAGAT-[BHQ1]-3′ (probe). A standard reaction mixture (50 µl) contained Taqman primer/probe combination, cDNA equivalent to 100 ng total RNA, and qPCRmix-HS master mix. The real-time PCR thermal conditions for all genes were 55°C for 5 min, 95°C for 10 min, followed by 40 cycles each at 95°C for 10 s and 57°C for 1 min (signal collection temperature). Relative quantitative analysis was carried out by comparing threshold cycle number for target genes and a reference β-actin mRNA, amplified in separate tubes.

### Subcellular protein fractionation and Western blot analysis

Huh7 cells were transfected with the plasmid coding HCV protein or pcDNA3.1(+) plasmid. 36 hours after transfection the cells were harvested and washed twice in PBS. The cytoplasmic and nuclear fractions were obtained by NE-PER kit according to manufacturing protocol. Western analysis was performed as described above. Membranes were probed with primary antibody: anti-Nrf2 antibody (5 µg/ml), anti-HO1 antibody (2 µg/ml), anti-Nqo1 antibody (1 µg/ml), anti-actin antibody (1 µg/ml). We used ECL Pico Substrate (Thermo Scientific) for detection.

### Statistical analysis

Statistical analysis was performed with BioStat 2008 software (AnalystSoft, Vancouver, Canada). All data are presented as means±SD. Differences between two groups were compared using paired Student's *t*-test. For comparison between multiple groups, ANOVA followed with Tukey-Kramer post test was applied. A value p<0.01 was considered as statistically significant.

## Supporting Information

Figure S1 **Immunoblot analysis of HCV protein expression in Huh7 cells 36 h posttransfection.**
(TIF)

Figure S2 **Analysis of protein expression kinetics and of their influence in ROS production and ARE-luciferase expression.** (**A**) Accumulation of core, NS5A, and NS5B proteins in Huh7 cells. (**B**) Kinetics of accumulation of reactive oxygen species (ROS) in cells expressing NS5A or core proteins. (**C**) Time-course of ARE-dependent luciferase expression in cells treated with tBHQ or expressing NS5A or core proteins. tBHQ was added at the time point of 18 h posttransfection of the NS5A-expressing cells. NS5B protein-expressing cells were used as a negative control.
(TIF)

Figure S3 **Characterization of the constructed ARE-luciferase reporter plasmid.** tBHQ induces ARE-luciferase expression and shown no notable cytotoxicity. The Huh7 cells were transfected with ARE-luciferase reporter, treated with 100 µM tBHQ or 400 µM H_2_O_2_, and luciferase activity was quantified 10 h later (**A**). Cytotoxicity was measured by standard MTT test (**B**). Error bars indicate SD. *P<0.01 versus DMSO (Tukey-Kramer test).
(TIF)

Figure S4 **Expression of core protein activates both ARE-luciferase (B) and SV40-luciferase (A,B) activity.** The effect on ARE-luciferase is inhibited by PDTC and therefore is partially mediated by ROS (**B**). Error bars indicate SD. *P<0.01 versus DMSO (Tukey-Kramer test).
(TIF)

Figure S5 **HCV proteins induce expression of HO-1 and Nqo1 genes 31 h posttransfection.** mRNA levels of the respective genes were measured by RT-qPCR. B-actin was used as an internal control. Error bars indicate SD.
(TIF)

Figure S6 **Influence of protein kinases inhibitors on ARE-dependent luciferase expression induced by HCV proteins.** HCV proteins induce ARE-luciferase via PKC in a ROS-dependent, and by PI3K and CK2 in a ROS-independent manner. Huh7 cells were cotransfected with pP-ARE luciferase reporter and plasmids expressing individual HCV proteins, treated with the inhibitors of PI3K (wortmannin, Wo), p38 (SB 239063, Sb), ERK1/2 (PD98,059, Pd), CK2 (DRB), or PKC (Ro 31-8220, Ro), and luciferase activity was measured. Error bars indicate SD.
(TIF)
