# Supplementary figures and images for: Hepatitis C Virus Proteins Activate NRF2/ARE Pathway by Distinct ROS-Dependent and Independent Mechanisms in HUH7 Cells
Source: PLoS One. 2011 Sep 13;6(9):e24957. doi: 10.1371/journal.pone.0024957 (PMC3172309; doi:10.1371/journal.pone.0024957)

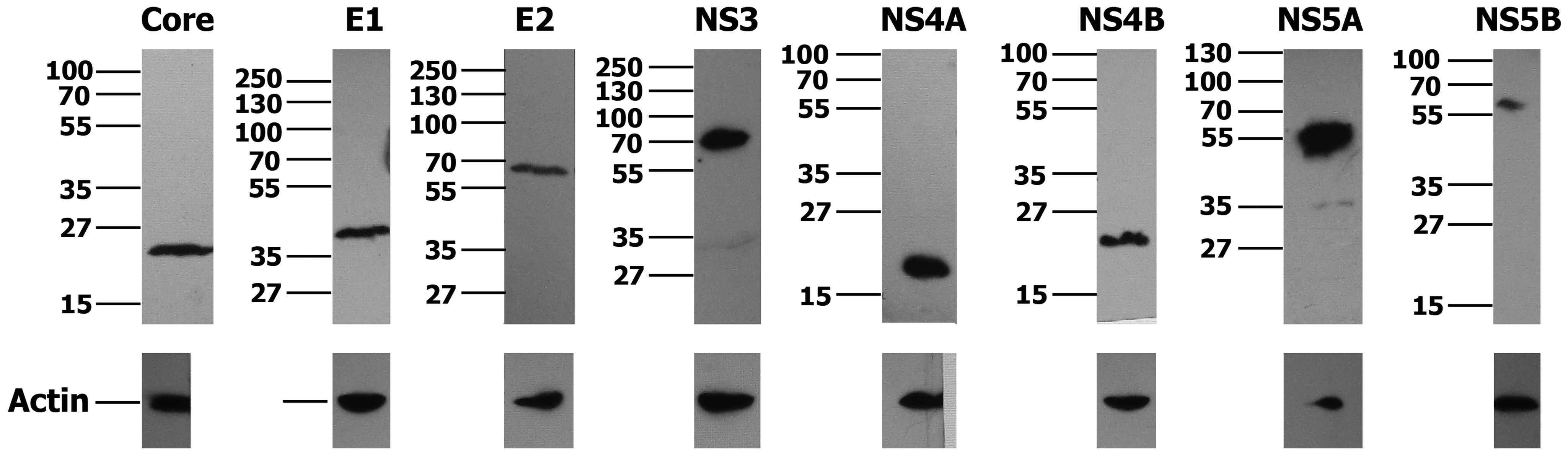

Supplement: Figure S1 — Immunoblot analysis of HCV protein expression in Huh7 cells 36 h posttransfection. (TIF) [file pone.0024957.s001.tif]

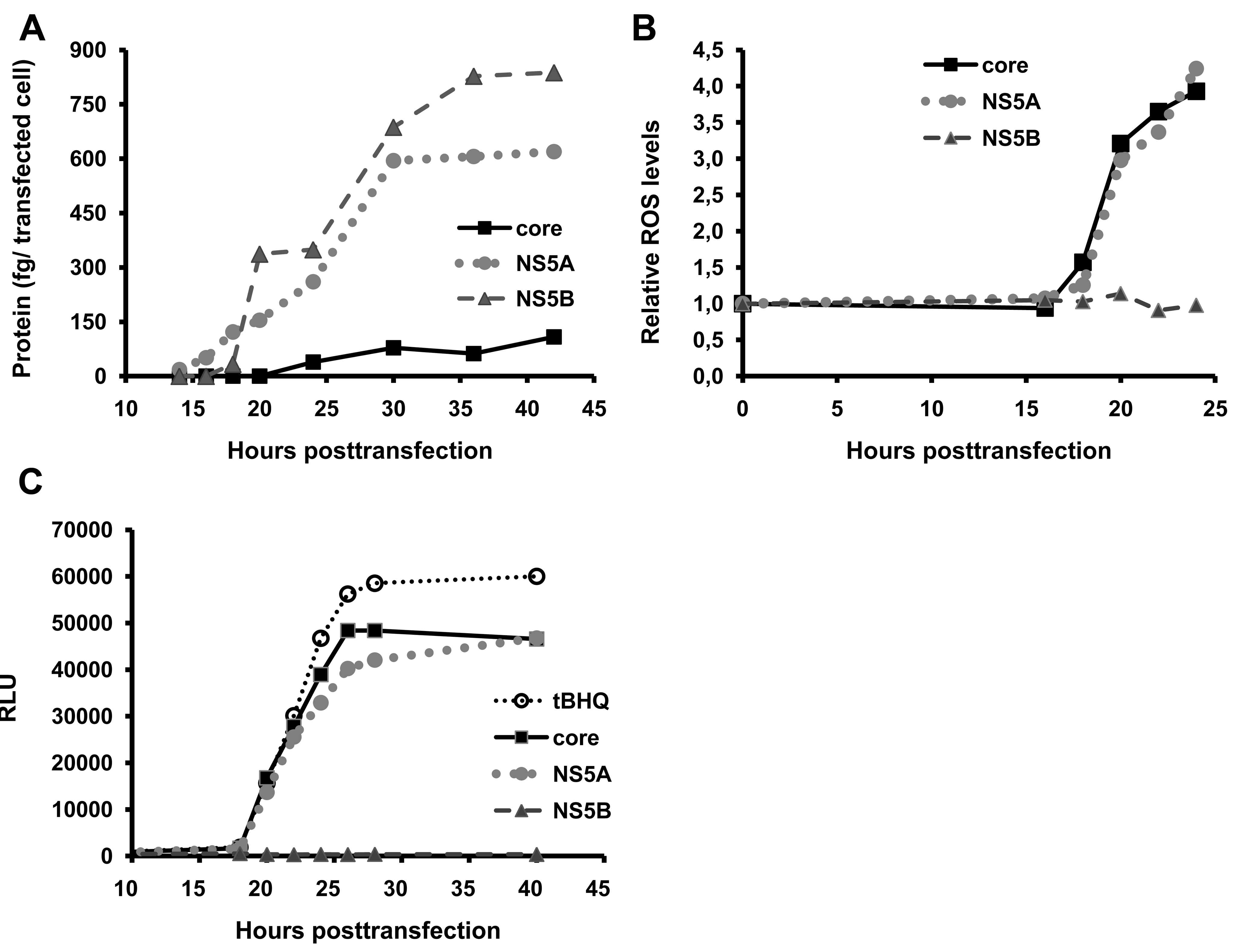

Supplement: Figure S2 — Analysis of protein expression kinetics and of their influence in ROS production and ARE-luciferase expression. (A) Accumulation of core, NS5A, and NS5B proteins in Huh7 cells. (B) Kinetics of accumulation of reactive oxygen species (ROS) in cells expressing NS5A or core proteins. (C) Time-course of ARE-dependent luciferase expression in cells treated with tBHQ or expressing NS5A or core proteins. tBHQ was added at the time point of 18 h posttransfection of the NS5A-expressing cells. NS5B protein-expressing cells were used as a negative control. (TIF) [file pone.0024957.s002.tif]

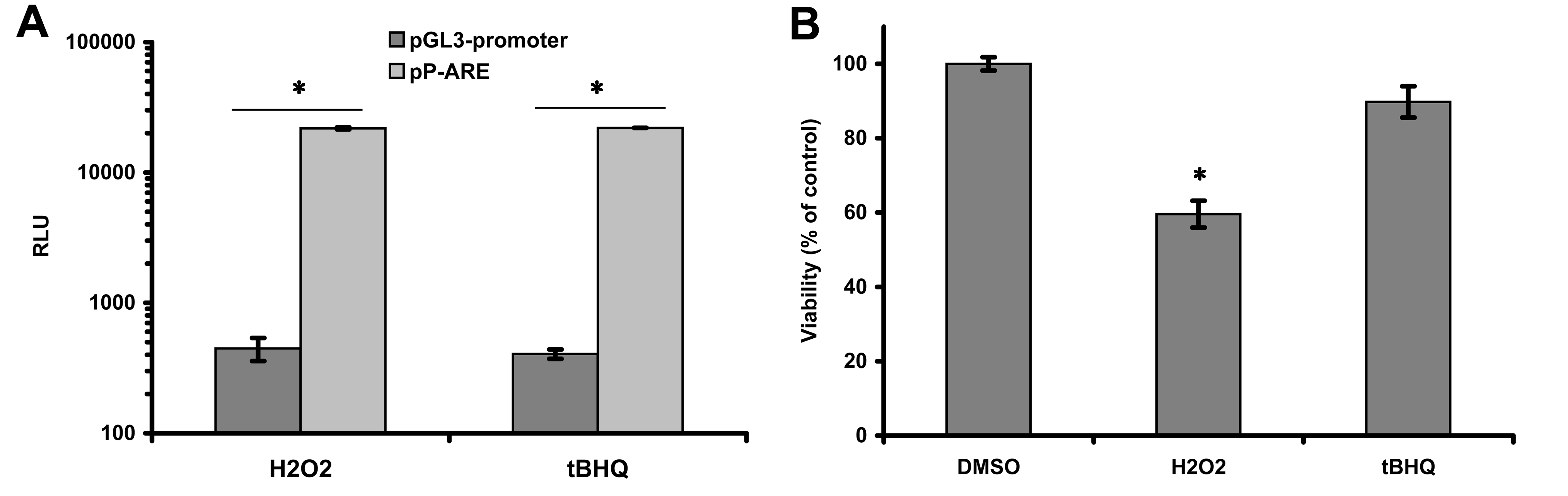

Supplement: Figure S3 — Characterization of the constructed ARE-luciferase reporter plasmid. tBHQ induces ARE-luciferase expression and shown no notable cytotoxicity. The Huh7 cells were transfected with ARE-luciferase reporter, treated with 100 µM tBHQ or 400 µM H2O2, and luciferase activity was quantified 10 h later (A). Cytotoxicity was measured by standard MTT test (B). Error bars indicate SD. *P<0.01 versus DMSO (Tukey-Kramer test). (TIF) [file pone.0024957.s003.tif]

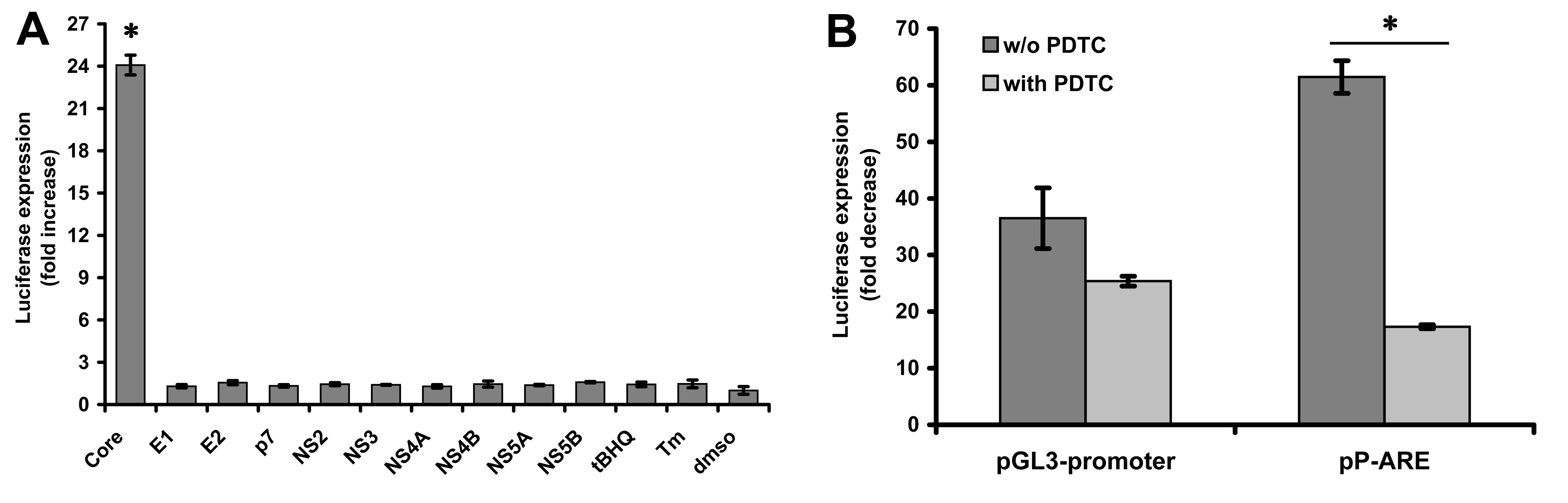

Supplement: Figure S4 — Expression of core protein activates both ARE-luciferase (B) and SV40-luciferase (A,B) activity. The effect on ARE-luciferase is inhibited by PDTC and therefore is partially mediated by ROS (B). Error bars indicate SD. *P<0.01 versus DMSO (Tukey-Kramer test). (TIF) [file pone.0024957.s004.tif]

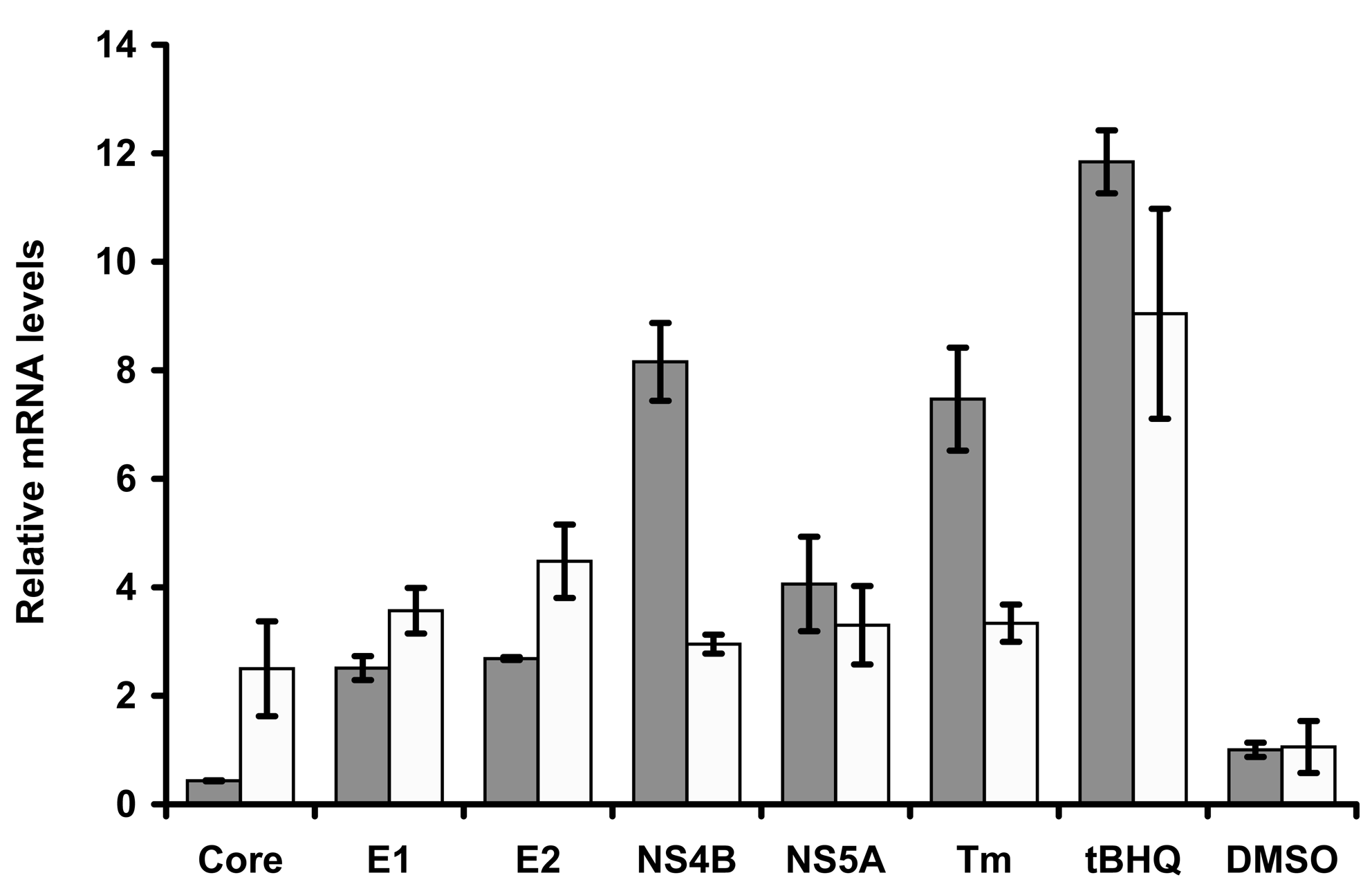

Supplement: Figure S5 — HCV proteins induce expression of HO-1 and Nqo1 genes 31 h posttransfection. mRNA levels of the respective genes were measured by RT-qPCR. B-actin was used as an internal control. Error bars indicate SD. (TIF) [file pone.0024957.s005.tif]

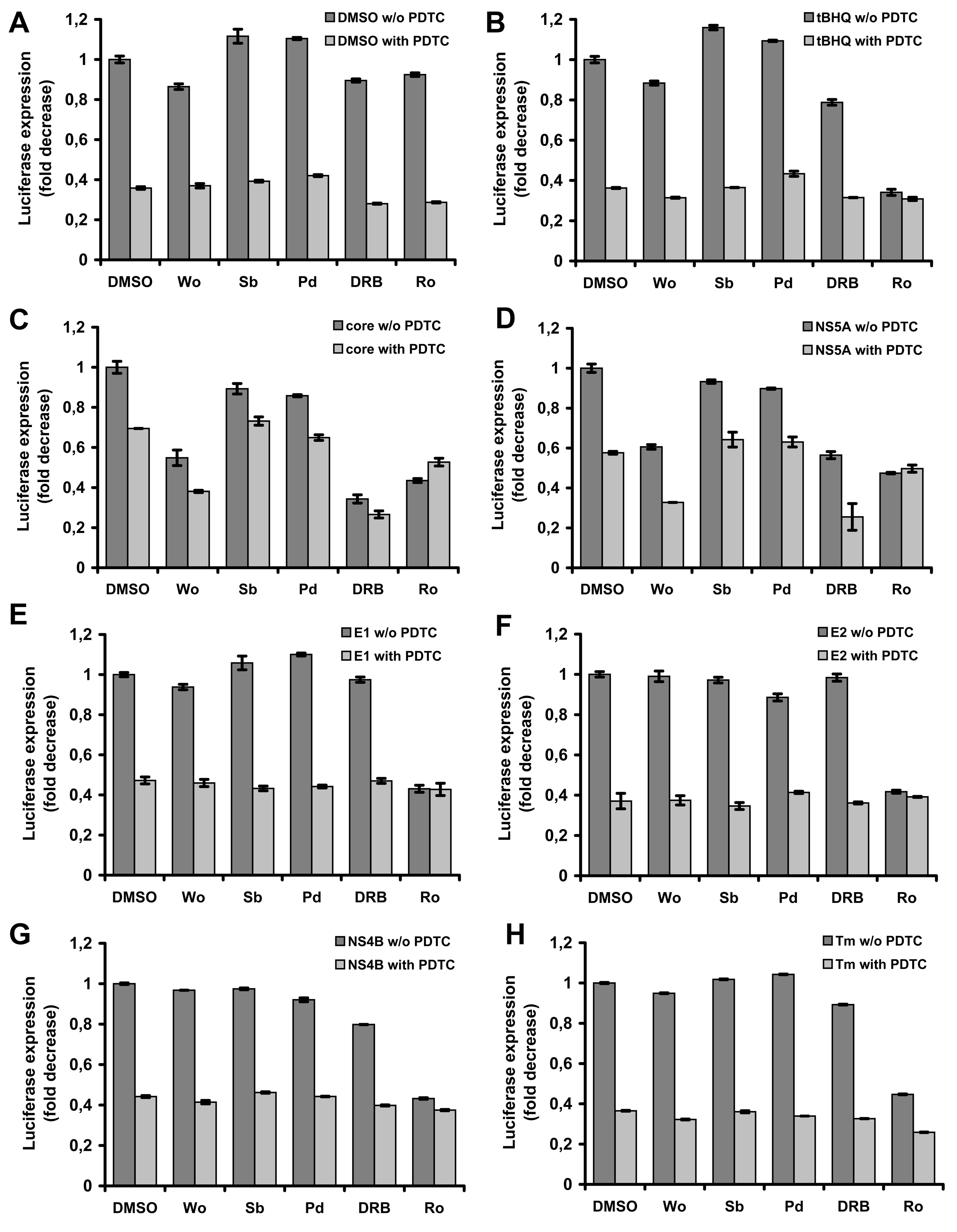

Supplement: Figure S6 — Influence of protein kinases inhibitors on ARE-dependent luciferase expression induced by HCV proteins. HCV proteins induce ARE-luciferase via PKC in a ROS-dependent, and by PI3K and CK2 in a ROS-independent manner. Huh7 cells were cotransfected with pP-ARE luciferase reporter and plasmids expressing individual HCV proteins, treated with the inhibitors of PI3K (wortmannin, Wo), p38 (SB 239063, Sb), ERK1/2 (PD98,059, Pd), CK2 (DRB), or PKC (Ro 31-8220, Ro), and luciferase activity was measured. Error bars indicate SD. (TIF) [file pone.0024957.s006.tif]
